# Geometric Attributes of Retaining Glycosyltransferase Enzymes Favor an Orthogonal Mechanism

**DOI:** 10.1371/journal.pone.0071077

**Published:** 2013-08-01

**Authors:** Brock Schuman, Stephen V. Evans, Thomas M. Fyles

**Affiliations:** 1 Department of Biochemistry and Microbiology, University of Victoria, Victoria, British Columbia, Canada; 2 Department of Chemistry, University of Victoria, Victoria, British Columbia, Canada; Russian Academy of Sciences, Institute for Biological Instrumentation, Russian Federation

## Abstract

Retaining glycosyltransferase enzymes retain the stereochemistry of the donor glycosidic linkage after transfer to an acceptor molecule. The mechanism these enzymes utilize to achieve retention of the anomeric stereochemistry has been a matter of much debate. Re-analysis of previously released structural data from retaining and inverting glycosyltransferases allows competing mechanistic proposals to be evaluated. The binding of metal-nucleotide-sugars between inverting and retaining enzymes is conformationally unique and requires the donor substrate to occupy two different orientations in the two types of glycosyltransferases. The available structures of retaining glycosyltransferases lack appropriately positioned enzymatic dipolar residues to initiate or stabilize the intermediates of a dissociative mechanism. Further, available structures show that the acceptor nucleophile and anomeric carbon of the donor sugar are in close proximity. Structural features support orthogonal (front-side) attack from a position lying ≤90° from the C1-O phosphate bond for retaining enzymes. These structural conclusions are consistent with the geometric conclusions of recent kinetic and computational studies.

## Introduction

Understanding the fundamental structure-function relationships of glycosyltransferase enzymes is an essential step in the directed development new drugable inhibitors. Glycosyltransferases synthesize biological oligo- and polysaccharides, many of which have been associated with disease processes. In addition to being the defective product of a number of human genetic disorders *e.g.* [1]–[3], glycosyltransferases play critical roles in many facets of infection (*e.g.* [4]–[10]), immunity (*e.g.* [11]–[13]) and cancer (*e.g.* [14]–[22]. Despite the potential of these targets, the underlying biochemical mechanism of the glycosyltransferases is still poorly understood and is hampering focussed drug development.

**Figure 1:**
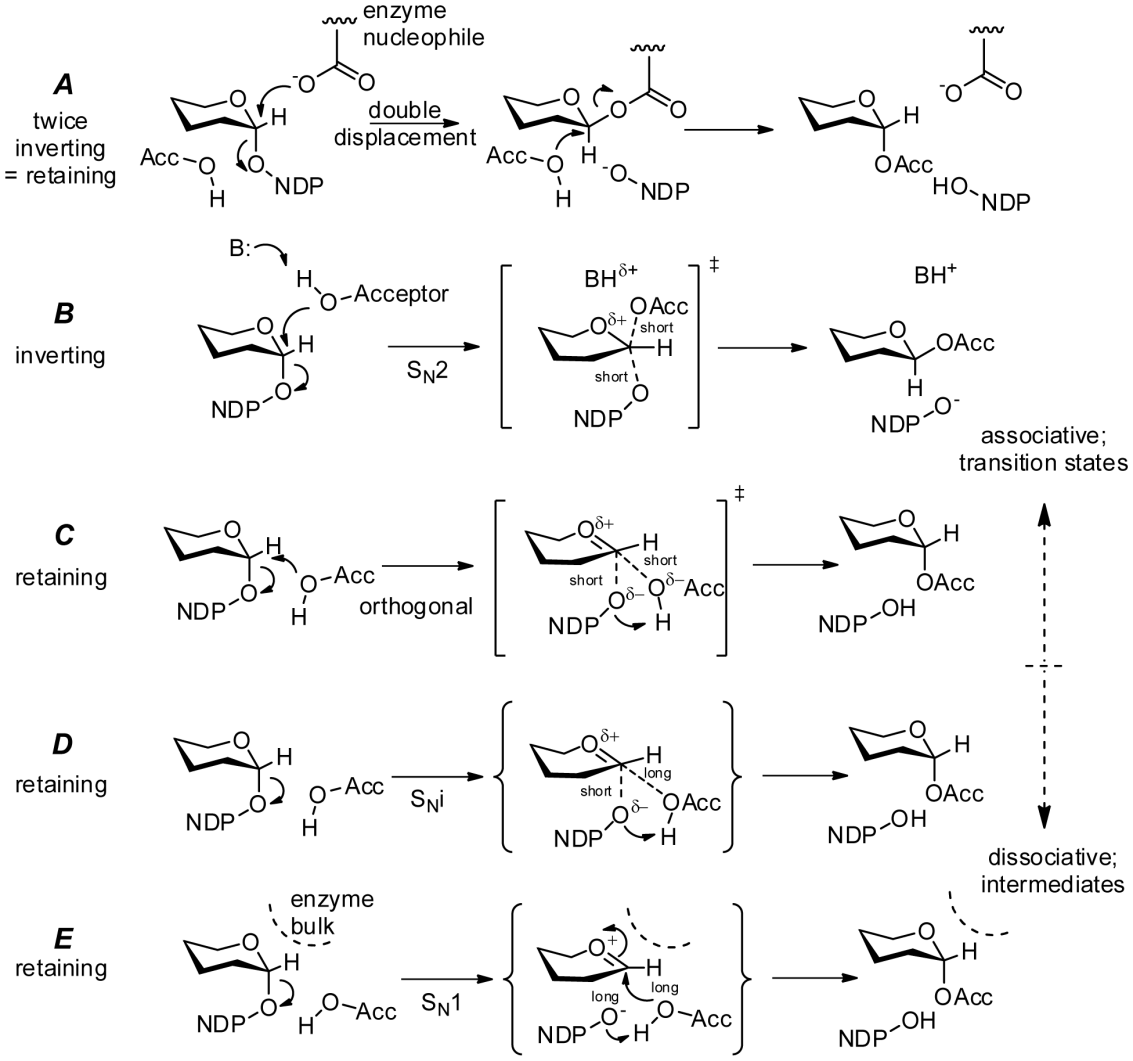
Proposed glycosyltransferase mechanisms. (**A**)A double displacement mechanism utilizing two inversions with net retention of stereochemistry involving a covalent glycosyl-enzyme intermediate. The individual steps are inverting via (**B**) an S_N_2 process. Inverting Leloir glycosyltransferases promote a backside nucleophilic attack on C1 by the acceptor from an inline (usually equatorial) position, with resulting inversion of the anomeric bond stereochemistry. Alternative mechanisms for retaining glycosyltransferases include: (**C**) an orthogonal mechanism consisting of nucleophilic attack on C1 by the acceptor concurrent with leaving group loss from a position approximately at right angles to the C1-leaving group axis; (**D**) an S*_N_*i mechanism involving an intermediate with oxocarbenium character followed by rapid internal nucleophilic attach by the acceptor nucleophile; or (**E**) an S*_N_*1 mechanism involving a discreet oxocarbenium intermediate. All mechanisms require proton transfers of the hydroxyl hydrogen of the acceptor to an enzymatic baseor the departing leaving group.

Leloir glycosyltransferases donate a monosaccharide unit from a nucleotide-sugar (“glycosyl donor”) to a “glycosyl acceptor”, typically a hydroxyl group of an oligosaccharide [23]. Two stereochemical classes are known. Retaining glycosyltransferase enzymes preserve the stereochemistry about the anomeric carbon atom of the donor sugarin the new glycosidic linkage i.e. an axial donor stereochemistry results in an axial stereochemistry in the product. Inverting glycosyltransferases invert the anomeric stereochemistry i.e. an axial donor becomes equatorial in the product. The mechanism of the inverting reaction is widely accepted and is mechanistically straightforward; the acceptor hydroxyl acts as a nucleophile and approaches the anomeric carbon from the opposite side to the donor-nucleoside linkage eventually resulting in inversion of anomeric stereochemistry as the nucleoside leaves. The mechanism for retaining glycosyl transfer stereospecificity is more problematic and remains a matter of debate. Mechanisms can be broadly classified as proceeding with primarily dissociative (S*_N_*1) or primarily associative (S*_N_*2)character. Postulate mechanisms are outlined in **Figure 1**.

The earliest mechanism proposed to explain retaining glycosyltransfer was the double displacement mechanism (**Fig. 1A**): an initial nucleophilic substitution provided by an enzyme nucleophile forms an inverted covalent enzyme-carbohydrate intermediate which is in turn attacked by the acceptor molecule leading to a net retention of anomeric stereochemistry [24]. Each step of this process occurs as described above for inverting glycosyltransferases via backside attack through a single transition state resulting in inversion (**Fig. 1B**). Although there is mass spectrometric evidence that migh support the existence of a covalent glycosyl-enzyme intermediate in retaining glycosyltransferases [25], [26], such an intermediate has not been detected structurally, kinetically or spectroscopically [27]–[33].

Retaining substitution with dissociative character (**Fig. 1D,E**) has been proposed as an alternative [27]–[33]. Nucleotide diphosphates (NDPs) are excellent leaving groups, and the resulting oxocarbenium cation could be stabilized by adjacent protein dipoles. Both of these factors would favour a dissociative process. However, unimolecular dissociation would result in the loss of stereochemical integrity. No partially inverted products have ever been described for a retaining glycosyltransferases. Enzymes that do involve dissociative character are hydrolases or transferases that do not transfer stereocenters (reviewed in [34]). Thus proposed dissociative pathways also require that steric hindrance is provided by the enzyme to force the generation of retained product (**Fig. 1E**).

Another dissociative variant is called S*_N_*i (*N*ucelophilic *S*ubstitution with *i*nternal return; **Fig. 1D**). This mechanism involves partial nucleotide diphosphate dissociation and charge development within a polar active site cage prior to nucleophilic attack by the acceptor [35]. This is usually drawn as a dissociative transition state with a long distance interaction between the anomeric carbon atom and the incoming nucleophile and a shorter interaction with the departing leaving group. This leads to a short-lived intermediate ion pair which rapidly collapses in a second step. S*_N_*i has previously been invoked to explain gas-phase chemical reactions, butits acceptance as a suitable pathway for retaining glycosyltransferases has met with resistance [25], [26].

Recent kinetic investigation of trehalose-6-phosphate synthase, a metal-free retaining glycosyltransferase, concludes that the available evidence favors a “front-side S_N_i” intermediate having substantial dissociative character at the rate-limiting transition state [36]. The same conclusion is supported by kinetic and computational studies of the solvolysis of isotopically labeled α-D-glycopyranosyl fluorides in hexafluoro-2-propanol in which the kinetic isotope effects are most consistent with a “front-face” geometry [37]; the authors favor a stepwise S_N_i intermediate, although the data show a concerted transition-state with both the leaving group and the incoming nucleophile in close proximity to the anomeric carbon gives closely similar computed isotope effects. Finally, a computational study of lipopolysaccharyl-α-1,4-galactosyltransferase C, a Leloir retaining glycosyltransferase finds a front-side geometry at the transition state which is described as “S_N_i-like” with significant charge development in the donor sugar as the transition state is reached [38].

It has also been suggested that retaining transfer may contain both dissociative and associative elements [39], and the two are not mutually exclusive. Absolute distinction between associative and dissociative reaction pathways is not always possible; dissociative pathways progress into associative pathways as the transition state develops a less stable and shorter-lived oxocarbenium cation intermediate [40]; the mechanism illustrated as **Fig. 1C** reflects this continuum from the S_N_i mechanism of **Fig. 1D**, involving a discrete if short-lived intermediate, to the S_N_2 case of **Fig. 1B** involving only a single transition state without an intermediate. The precise character of the **Fig. 1C** mechanism depends upon the intimate details of charge development and nucleophilic attack (*vide infra*). We use the term “orthogonal” for this mechanism to mean a process involving the nucleophile and the leaving group on the same side (a.k.a. “front-side” attack) where the approach of the nucleophile is approximately orthogonal to the breaking bond axis, and proceeding in a single step from reactants to products without an intermediate.

Knowledge of the mechanistic details of glycosyltransferases can be derived from a number of experimental approaches of which structural studies play a central role in providing starting points for computation, and geometrical constraints on the enzymatic groups required to interpret the kinetics. The modest degree of sequence homology among glycosyltransferase families has made the prediction of tertiary structures difficult. However, structural determinations in recent years have revealed that the catalytic domains of most glycosyltransferases display one of two fold types designated GT-A or GT-B [41], [42]. With few exceptions, the donor binding Rossmann folds of glycosyltransferases contain a “DXD motif” that consists of an Asp-X-Asp amino acid triplet used to coordinate the phosphates of the donor molecule through a divalent cation with octahedral geometry. Some inverting enzymes do not require a divalent metal cofactor, though to date there is only one retaining Leloir-type enzyme that has been characterized as metal independent [43].

A neutron structure of the human retaining enzyme GTA at LANCE PCS (PDB 4DHH associated with [44]) has been reported. More detailed analysis of this structure has revealed an aprotic active site that appears to be incompatible with a dissociative mechanism. To examine the generality of this observation, we report a re-investigation of the published geometric presentation between donor and acceptor substrates in the enzymatic active sites of previously reported GT-A fold glycosyltransferases. The analysis of the structures, together with literature data from NMR, MS, kinetics, and computational studies,point to the orthogonal mechanism for retaining glycosyltransferases as both the simplest and the most consistent with the available data.

## Methods

Deposited GT-A fold PDBs identified by CAZy were analyzed for geometric parameters using SetoRibbon,a continued development of SETOR [45] with adaptations for high throughput geometric analysis. Of the eleven families with deposited structures (**Table 1**), four were found which had unambiguous densities complete for donor (nucleotide and monosaccharide) and acceptor molecules (or analogs): GT-6 retaining enzyme human blood-group A glycosyltransferase, GTA (L266M/G268A [46]); GT-7 inverting enzyme 1,4-galactosymtransferase T1, GalT1 (wt [47]); GT-8 retaining enzyme lipooligosaccaride transferase C, LgtC (C128/174S [48]); and GT-43 inverting enzyme β-1,3-glucuronosyl transferase 1, GlcAT-1 (M344H [49], [50]). Retaining enzymes GTA and LgtC were both crystallized with deoxy-acceptor analogs, which allowed confident modeling of their respective nucleophilic atoms. Inverting enzymes GlcAT-I and GalT1 required combining separate donor-bound and acceptor-bound structures for analysis, and the accuracy of the analyzed geometry is potentially reduced as the same steric constrains as the bisubstrate liganded active site are not necessarily applicable. For example unambiguous density for the carbohydrate moiety of retaining enzyme GTA mutants have been observed in 4 distinct conformations [46], [51], though all but one conformation is incompatible with catalytic turnover. Structures have been deposited of the two retaining enzymes with both donor and acceptor analogs bound simultaneously, where the deviation of the analogs from positions observed occupied by the natural acceptors is only ∼0.1 Å.

**Table 1:** GT-A fold glycosyltransferase families with deposited structures.

Family	Example enzyme	Stereo-specificity	Example Complex(es)
GT-2	SpsA	Inverting	UDP
**GT-6**	**GTA**	**Retaining**	**UDP-Gal+Gal-Fuc**
**GT-7**	**GalT1**	**Inverting**	**UDP-Gal, GlcNAc-GlcNAc**
**GT-8**	**LgtC**	**Retaining**	**UDP-Gal+Gal-Glc**
*GT-13*	*GnT1*	*Inverting*	*UDP-GalNAc, UDP-Glc*
GT-15	Kre2	Retaining	GDP+Man+GlcNAc
GT-27	GNAc:Pep	Retaining	UDP+GlcNAc
**GT-43**	**GlcAT-I**	**Inverting**	**UDP-GlcUA, UDP+Gal-Gal-Xyl**
*GT-55*	*MpgS*	*Retaining*	*GDP-Man*
*GT-64*	*Extl2*	*Retaining*	*UDP-GalNAc*
GT-78	MgS	Retaining	GDP
GT-81	ManT	Retaining	GDP

Bold underlined families were assessed to have unambiguous whole acceptor and donor molecule electron density for analysis; those in italics have donor density.

**Figure 2:**
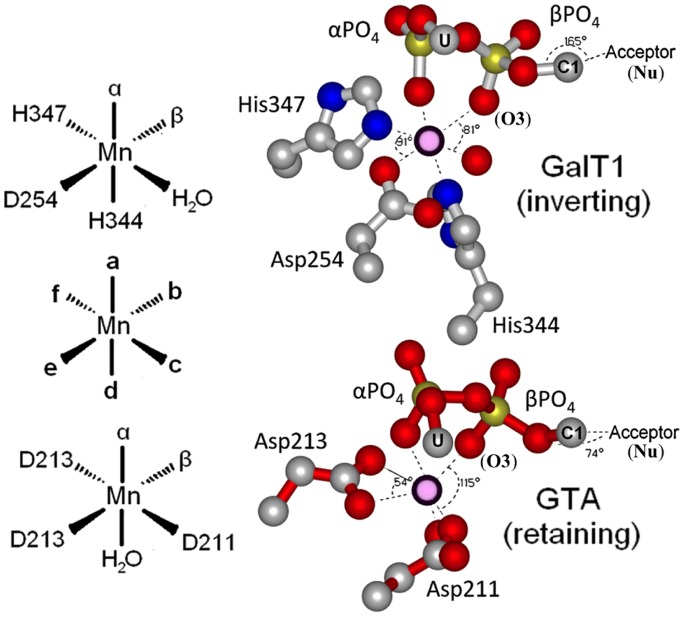
The a-f nomenclature used to describe octahedral binding partners. Inverting enzymes such as GalT1 (top) achieve nearly perfect octahedral geometry about the coordinated metal ion (displayed angles of 81° and 91° compared to ideal octahedral 90° bond angles) with subsequent “inline” (approaching 180°) placement of the acceptor nucleophile for classic inverting S*_N_*2 backside attack. Retaining enzymes such as GTA (bottom), however, use an arrangement of acidic residues, often with acute bidentate Asp coordination, which severely skews metal geometry (displayed angles of 54° and 115°) and allots sufficient room between phosphate oxygens for orthogonal attack from the acceptor. **U** is uridine, **C1** is donor galactose **C1**.

The geometry between the phosphates and acidic residues that coordinate the divalent metal cofactor **(M)** were determined with SetoRibbon, a continued development of SETOR [45] with adaptations for high throughput geometric analysis. The molecular geometry surrounding the metal is roughly octahedral, though when very acute bidentate Asp coordination is employed this can be skewed to nearly trigonal prismatic dimensions. For consistency we have labeled the α-phosphate **O2** as **a** at the apex of the coordination octahedron, and place the second β-phosphate **O1** as **b** in the clockwise position of the projection with the remaining coordinating atoms labeled **c-f** as illustrated in **Figure 2**. The metal cation is the focal point of the alignments analyzed. Instead of optimizing RMS for the polypeptide chain, alignments were made using metal cation **M**, α-phosphate **O1** and β-phosphate **O2** as fixed positions from which to compute relative distances and angles.

**Figure 3:**
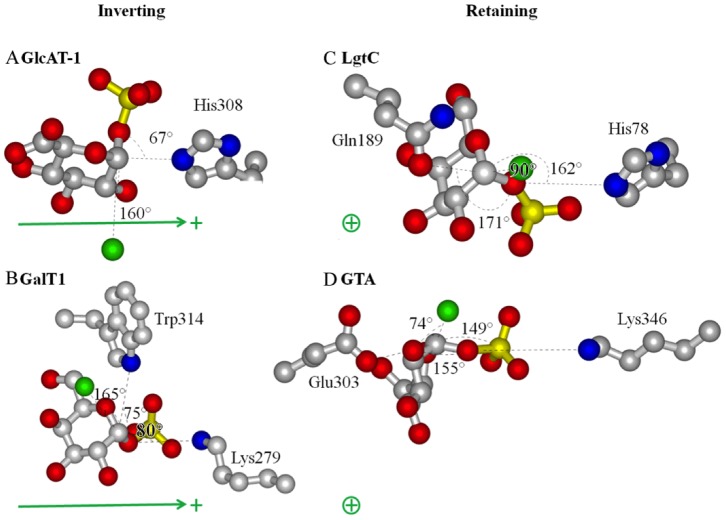
Reaction center dipoles. Opposed to the placement of the acceptor nucleophile (Green spheres), the closest polar residues to leaving group β-phosphate **O3** and **C1** lay acutely (67° and 75°, respectively) for inverting enzymes (**A,B**) and lie nearly in-line (171° and 155°, respectively) for retaining enzymes GTA (**C,D**). This may help to stabilize the associative intermediates without hindering the opposite angle of attack from the acceptor molecule nucleophile. Also, the **O3**– **C1**vectors lay looselyperpendicular to the enzyme macrodipole vectors to stabilize the inverting transition states (green arrows) (**A,B**), and loosely parallel to stabilize the retaining transition states (**C,D**) (green ⊕, dipole oriented with the cationic end above the page and the anionic end in the page).

Geometric parameters that pertain to the discussion of the mechanism include the nucleophilic distance from the acceptor nucleophilic oxygen atom **Nu** to the donor monosaccharide electrophilic center (**C1** for all of the enzymes analyzed),the angle between the incoming nucleophile and leaving group β-phosphate oxygen **O3**, the distance between **Nu** and **O3**, the distance to the closest enzyme electronegative atom observed for **O3** and **C1**, the angle between **O3** and **C1** nearest dipole vectors, and the angles between donor β-phosphate **O1** and the adjacent coordinating acidic ligands of the metal ion. These are listed in **Table 1**. Protein macrodipoles (represented in **Figure 3**) were estimated with the Protein Dipole Moments Server [52]and compared to the path between **Nu** and **C1**.

## Results and Discussion

### Analysis of Available Structural Data

It is well accepted that dissociative (S*_N_*1 or S_N_i) mechanisms require activation and stabilization of the ions as they form [53]–[55]. In homogenous solution this is usually achieved by a polar protic solvent such as water. Within an enzyme, solvent molecules are thought to be excluded from the proximity to the donor sugar anomeric carbon **(C1)** electrophile of these enzymes to prevent destructive donor hydrolysis from nucleophilic attack by water. The neutron structure of GTA shows no water in proximity to the active site; indeed, the active site is aprotic to a distance of >4.5 Å from the reaction site. In the absence of solvent it falls upon the enzyme to provide correctly positioned and oriented dipoles with which to stabilize anion pair intermediates. The closest observed enzymatic polar groups to donor sugar **C1**, β-phosphate nucleofuge (atom **O3**) and ring **O5** for a number of retaining and inverting enzymes are outlined in **Table 2**. These centers would share the charge of an intermediate oxocarbeniumion as would develop in a S_N_1 or S_N_i process. All lie too far away (∼4.5 Å) to initiate a dissociative mechanism, but may extend the lifetime of a dipola rtransition state.

**Table 2:** Active site residue identities and geometric values.

Stereospecificity	Inverting			Retaining			
Example enzyme	GlcAT-I	GalT1	*GnT1*	LgtC	GTA	*Extl2*	*ManT*
PDB(1)	1V84	1TVY	2AM3	1GA8	2RJ7	1OMZ	2WVL
PDB(2)	1KWS	1TW5					
Nu – C1 dist.	4.4 Å	4.2 Å	4.0 Å^a^	2.2 Å	2.5 Å		
<Nu-C1-O3	160°	165°	151°^a^	90°	74°	NA	NA
Nu – O3 dist.	5.8 Å	5.6 Å	5.4 Å^a^	2.8 Å	2.2 Å		
O3– nearest polar X	H_2_O^b^	K279	Y184	H78	K346	H_2_O^b^	Y268
O3- X dist.	4.4 Å	4.4 Å	5.4 Å	4.7 Å	5.6 Å	3.8 Å	4.4 Å
<X-O3-C1	91°	80°	87°	171°	149°	131°	59°
C1 nearest polar Y	H308	W314	D211	Q189	E303	R293	D167
C1-Y dist	3.6 Å	4.5 Å	5.2 Å	3.5 Å	4.8 Å	3.7 Å	3.5Å
<Y-C1-O3	67°	75°	71°	162°	155°	167°	142°
O5 nearest polar Z	R156	W314	D291	Q189	R352	R293	D168
O5-Z dist.	5.9 Å	3.4 Å	3.9 Å	4.2 Å	5.8 Å	3.2 Å	3.8 Å
<Z-O5-C1	113°	123°	96°	82°	83°	97°	68°
**c** c	H2O^b^	H2O^b^	H2O^b^	D103	D211	H2O^b^	NA
<**b**M**c** c	89°	82°	87°	105°	116°	101°	NA
**f** c	D196	H347	H2O^b^	D105	D213	H2O^b^	N313
<**b**M**f** c	114°	104°	95°	92°	90°	88°	82°

With the exception of the GTA neutron diffraction studies [44] hydrogen atoms are not directly observed, so distances are given between centers of non-hydrogen atoms. Italic enzyme names indicate the model did not contain an acceptor molecule.

a PDB 2AM3 has a glycerol molecule modeled as an acceptor.

b It is likely that the active species are not actually water molecules, but residues in disordered regions of the polypeptide.

c **b**, **c** and **f** are octahedral binding partners to the coordinated metal atom **M** as described in **Figure 2**.

For retaining enzymes GTA and ManT the closest nucleophiles to **C1** (Glu303 and Asp167, respectively) could be considered candidate nucleophiles for a double displacement reaction, however structurally conserved nucleophiles are absent in many reported retaining enzymes including LgtC and Extl2 which have respective Gln and Arg residues in this position. The closest polar groups to donor sugar **C1**, **O5** and phosphate **O3** vary considerably, can carry either positive or negative charges, and often their mutation does not inhibit catalysis (*eg*. [25]). Furthermore, glycosyl transfer still proceeds when **O5** is substituted with sulfur [56], and as such an intermediate thiocarbenium ion is unlikely to be stabilized to the same extent by donation from sulfur as in a regular oxocarbenium intermediate.

It is noteworthy that the active site architecture for proximal dipoles is conserved and distinct for retaining and inverting enzymes (**Fig. 3** **,** **Table 2**). For the inverting enzymes the **C1** bond to the leaving group **O3** lay at acute angles from adjacent polar residues (**Fig. 3A**: 67° to His308 N∈ of GlcAT-1; **Fig. 3B**: 80° to Lys279Nζ and 75° to Trp314 N∈ of GalT1). In retaining enzymes the corresponding angles are obtuse (**Fig. 3C** 162 to His78 N∈ of LgtC; **Fig 3D:** 155° to Lys 346 Nζ and 155° to Glu303 O∈ of GTA). Significantly, this positions the polar groups and enzyme macrodipoles that stabilize the retaining and inverting transition states to lie approximately orthogonal to each other (**Fig. 3**). The orientations of the protein macrodipoles are conserved among retaining enzymes where they lie roughly perpendicular to the nucleophile approach as expected for stabilization of developing partial cationic charge without influencing leaving group departure or nucleophilic attack. The macrodipoles of inverting enzymes are similarly conserved, but are oriented parallel to the line of nucleophile approach, oriented to assist such an attack.

Further, the proximity of nucleophile (**Nu**) and electrophile (**C1**) in the retaining enzymes places tight constraints on the extent of dissociation possible before nucleophilic approach becomes the dominant interaction. The position of acceptor nucleophiles modeled from deoxy-acceptor crystal structures are observed at distances much less than 3 Å (2.5 Å for GTA and 2.2 Å for LgtC) from donor **C1**, whereas they wouldbe expected to reside greater than 3 Å away to allow UDP dissociation prior to nucleophilic attack [57].The computed transition states for glucopyranosyl fluoride solvolyses place the acceptor oxygen 3.02Å from **C1** in the S_N_i transition state and at 2.25 Å in the associative “front side” transition state [37]; the corresponding distance computed for the “S_N_i-like” transition state of a galactosyl transferase is 2.3 Å [38].**Nu** and **C1** can be much greater than 3 Å in a precatalytic conformation as is observed for both analyzed models of inverting enzymes (4.4 Å for GlcAT-I and 4.2 Å for GalT1).

**Figure 4:**
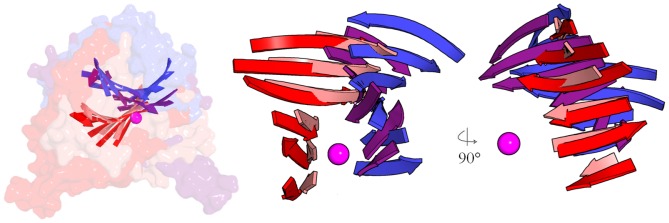
Retaining and inverting enzymes are entirely orthogonal. Theβ-sheets of the metal-nucleotide-sugar binding GT-A foldsof glycosyltransferase structures are superimposed by centering on the metal ion (magenta sphere) and the coordinated phosphates reveal that the general architecture of entire inverting or retaining enzymes are skewed by ∼90°. Color coding: purple, inverting GlcAT-I; blue, inverting GalT1; red, retaining GTA; pink, retaining LgtC. Left panel shows the superimposed solvent-accessible surfaces of the four structures with the folds embedded; the right panels isolate the β-sheets and show orthogonal perspectives.

Comparing the biologically active GTA structures to inverting enzymes such as galactosyltrasferasesβ4GalT1 reveals that the enzymes bind distinct metal-nucleotide-sugar conformers (**Figs. 2** **&** **3**), where the metal coordinating angle <**b-M-c** is less than <**b-M-f** for inverting enzymes and the opposite for retaining enzymes (**Table 2**). Inverting enzymes position **C1** for inline nucleophilic attack from the acceptor at an angle nearly 180° to the leaving group, while retaining enzymes position these groups at roughly 90° with respect to the **C1**-leaving group axis (**Fig. 2** 165° in inverting GalT1; 74° in retaining GTA). This is accomplished by inverting and retaining enzymes orienting their metal-nucleotide-sugar binding Rossmann folds approximately perpendicular to one another (**Figure 4**). Although geometrically distinct, in-line (inverting) and orthogonal transition states are not dissimilar; retaining enzymes apparently orient their acceptors to an apical position of a trigonal bipyramidal transition state with the leaving group occupying one of the equatorial positions. This orientation is formally accessible as a pseudo-rotation of the trigona bipyramidal geometry of the S*_N_*2 transition state, which is facilitated by structurally conserved obtusely oriented enzymatic dipoles for retaining enzymes, and is complemented by conserved acute dipoles for retaining enzymes (**Fig. 3**). Concurrent closing of the **Nu**-**C1** distance with leaving group loss and concurrent opening of the **H-C1-Nu** angle would result in associative retention of the donor’s anomeric stereochemistry (**Fig. 1C**). A similar reaction pathway has been suggested based on structural studies involving glycomimetic inhibitors [36], [58] and quantum chemical calculations [59], however the proposed mechanisms were still referred to as “S*_N_*i-like” implying the mechanism proceeds with a rate-limiting dissociative transition state and an intermediate of some finite lifetime.

**Analysis of other Published Data**

NMR analysis of donor hydrolysis facilitated by retaining glycosyltransferase enzymes in the absence of acceptor indicates that the cleaved monosaccharides are attacked by water from a retained position [59], [60]. This is inconsistent with a dissociative mechanism, as the steric constraints imparted by the enzyme’s fully liganded closed position would not be at play, with solvent molecules occupying both equatorial and axial positions.

Glycosyltransferases are bi-substrate enzymes and some mechanistic features can be inferred from the overall kinetic schemes observed. Double displacement should follow ping-pong kinetics as it develops a covalent intermediate, which can be identified on a Lineweaver-Burke plot as parallel lines at varied donor substrate concentrations; as is seen for trans-sialidase, for which such a mechanism has good precedent [61]. This is not observed for retaining glycosyltransferases such as MshA, assingle values for the acceptor K_M_ have been reported even when detailed bisubstrate Michaelis–Menten kinetic data has been collected [62].This kinetic evidence clearly does not support a 2-step mechanism with a covalent intermediate for this Leloir retaining glycosyltransferase.

The double displacement mechanism has strong precedent for enzymes that do not use metallic co-factors (reviewed in [34]) such as glycoside hydrolases, in which covalent glycosyl-enzyme intermediates have been trapped in crystal structures by using fluoridated substrates (*e.g.* [63], [64]). Such a species should be easier to trap for a glycosyltransferase as the strong donor leaving groups would leave a covalent sugar-enzyme intermediate in an energy well with the second attack being the rate limiting step [39], and there have been intensive attempts to trap such an intermediate. The only reports of enzyme-glycosyl intermediates have come from two independent ESI-MS studies, which identified apparent covalent intermediates using postulate nucleophile mutants [25], [26].One case showed the covalent species substituted remotely from the acceptor and produced at a rate much slower than enzymatic turnover, an observation of limited relevance to the catalytic mechanism. The other case showed the enzyme-glycosyl intermediate bound to the mutated cysteine; however, such an intermediate has not been observed by means other than MS.It has been suggested that these species could be the results of charged carbocation monosaccharides introduced in the gas phase by the electrospray conditions that undergo reaction with enzyme nucleophiles to produce such glycosylated species (reviewed in [65]). Kinetic isotope effect data [36] are also strong evidence against a stable covalent intermediate.

While double-displacement should follow ping-pong bi-substrate kinetics, S_N_i and associative mechanisms should follow either random associative or Theorell-Chance mechanisms. The latter is followed by a retaining galactosyl transferase [66]. The distinction between these is whether or not the ternary species builds to an extent that is kinetically significant. A developed S_N_i intermediate must avoid water attack, so a well-structured ternary complex formed in a random associative scheme is a reasonable possibility. There is no need for a long-lived ternary complex in an orthogonal mechanism. Thus the observation of Theorell-Chance kinetics is consistent with, but does not compel an orthogonal mechanism for the group transfer transition state.

### Mechanistic Proposal

The foregoing establishes that there is little direct evidence that Leloir retaining glycosyltransferases utilize a double-displacement or a fully developed S_N_1 mechanism (**Fig. 1A** or **E**). The focus therefore shifts to the dissociative pathway S_N_i and the orthogonal pathway (**Fig. 1D** or **C**). The distinction between an S*_N_i*and an orthogonal mechanism is found in the reaction profile and the timing of bond formation and bond breakage: if nucleophilic attack precedes or is concurrent with leaving group dissociation [59] with no enzymatic cage required to stabilize an oxocarbenium intermediate then there can be little dissociative character to the mechanism. The physical organic literature describes an associative mechanism as A_N_D_N_ indicating a single transition state with association of the nucleophile fully concurrent with departure of the leaving group. An alternative in which dissociation is slightly ahead of association would be D_N_A_N_ but in this case as well there is a single transition state without an intermediate. TheS_N_i pathway must involve an intermediate in a two-step process. It is described as D_N_*A_NSS_ or D_N_^‡^*A_NSS_ [66]with the notations denoting differing depths of the energetic well occupied by the intermediate of the two-step process.

**Figure 5:**
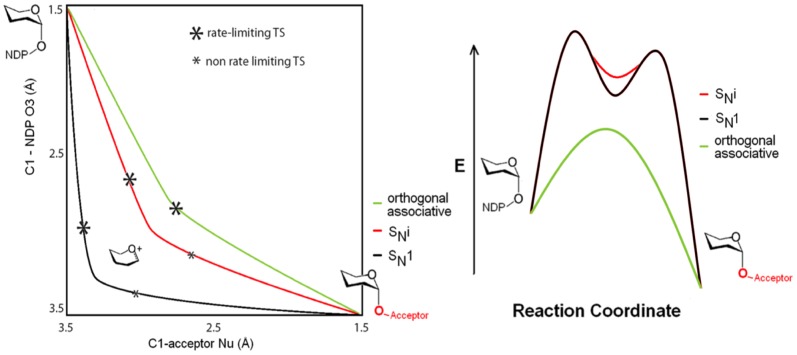
Geometries and energetics of mechanisms. (left) More O'Ferrall-Jencks plot illustrating the concurrent reaction coordinate geometry changes of proposed mechanisms. (right) Comparative reaction profile diagrams for dissociative (S*_N_*1, S*_N_i*) and orthogonal pathways. The relative energetics and rate-limiting transition state locations of the three pathways are speculative and are offset for clarity, but both S*_N_*1 and S*_N_i* displacement would certainly involve an intermediate in anenergy well whereas the orthogonal mechanism does not.

The available structural and kinetic data presented above are most consistent with an orthogonal mechanism of the D_N_A_N_ type. The assumed geometric and energetic consequences of the various relevant transition states and intermediates are sketched in **Figure 5** to visually highlight the distinctions. The geometrical changes of the alternative mechanisms are illustrated in the More O’Ferrall-Jencks diagram (**Fig. 5** **left**). The geometric consequences of the orthogonal mechanism are that the bond-making and bond-breaking phases are more closely coordinated than in the S_N_i trajectory. The energetic consequences are given in **Fig.5** **right** with the curves offset for clarity. The orthogonal mechanism involves a single barrier without intermediate, while the S_N_i and the more dissociative S_N_1reaction profiles involve and intermediate with two transition states. The geometric location of the transition states is indicated in **Fig.5** **left** with asterisks. The proposed orthogonal transition state likely lies close in energy to the transition state leading to a S_N_i intermediate. The key issue is that these pathways differ solely in the number of barriers and intermediates invoked. The D_N_A_N_ process we favour is in fact identical in energetic profile with the one determined computationally [38]. These authors described their trajectory as “S_N_i-like”. We disagree with this description as the trajectory does not involve an intermediate, so cannot be S_N_i by definition [66]; our use of “orthogonal” makes this distinction clearer.

## Conclusions

The foregoing structural and kinetic analyses are most consistent with an orthogonal pathway for glycosyltransferases that retain anomeric stereochemistry. From the structural perspective, retaining and inverting enzymes are observed to bind and to act upon distinct conformers of the metal nucleotide sugar complex. The donor substrate trajectory architecture observed for retaining enzymes is conserved so as to present the transferring monosaccharide anomeric electrophile from an orthogonal orientation. The distances observed between the approaching nucleophile and **C1** are too close to support full development of dissociation in the structures of both LgtC and GTA.

A double displacement mechanism requires an appropriately positioned and structurally conserved nucleophile in the active site. The active sites of many retaining enzymes do not contain well-positioned candidate nucleophiles, and those that have been proposed are often not sequentially or spatially conserved. In many cases, alanine mutagenesis of the proposed nucleophiles does not always abolish enzyme activity [26], [36].

Structural and kinetic evidence lies in favor of a single step orthogonal displacement. The substitution is positioned to initiate with nucleophilic attack and proceed through a trigonal bipyramidal transition state with incoming acceptor **Nu** axial and concurrently transferring a proton to an equatorial leaving β phosphate **O3**. This makes **C1** the focal point for a pseudorotation that pivots **O3** towards axial and **Nu** towards equatorial for retention (**Fig. 1C**). This mechanism provides the shortest physical route to glycosyltransfer (**Fig. 5**), avoiding energy wells and intermediates that have been elusive to detection. An orthogonal process avoids generation of even a short-lived oxocarbenium intermediate; it is therefore the simplest of the alternatives and the only one consistent with all available evidence.
